# Supplementary material for: Conditional chemoconnectomics (cCCTomics) as a strategy for efficient and conditional targeting of chemical transmission
Source: eLife. 2024 Apr 30;12:RP91927. doi: 10.7554/eLife.91927 (PMC11060718; doi:10.7554/eLife.91927)
Supplement: Figure 3—source data 3. [file elife-91927-fig3-data3.zip › Figure 3 source data3 part 1.zip!/Figure 3-Source data 3-part1/1071f2-1-CRISPResso_Report_ztwktvfp.zip!/CRISPResso_on_1071f2_2/1c.Alignment_barplot.pdf]

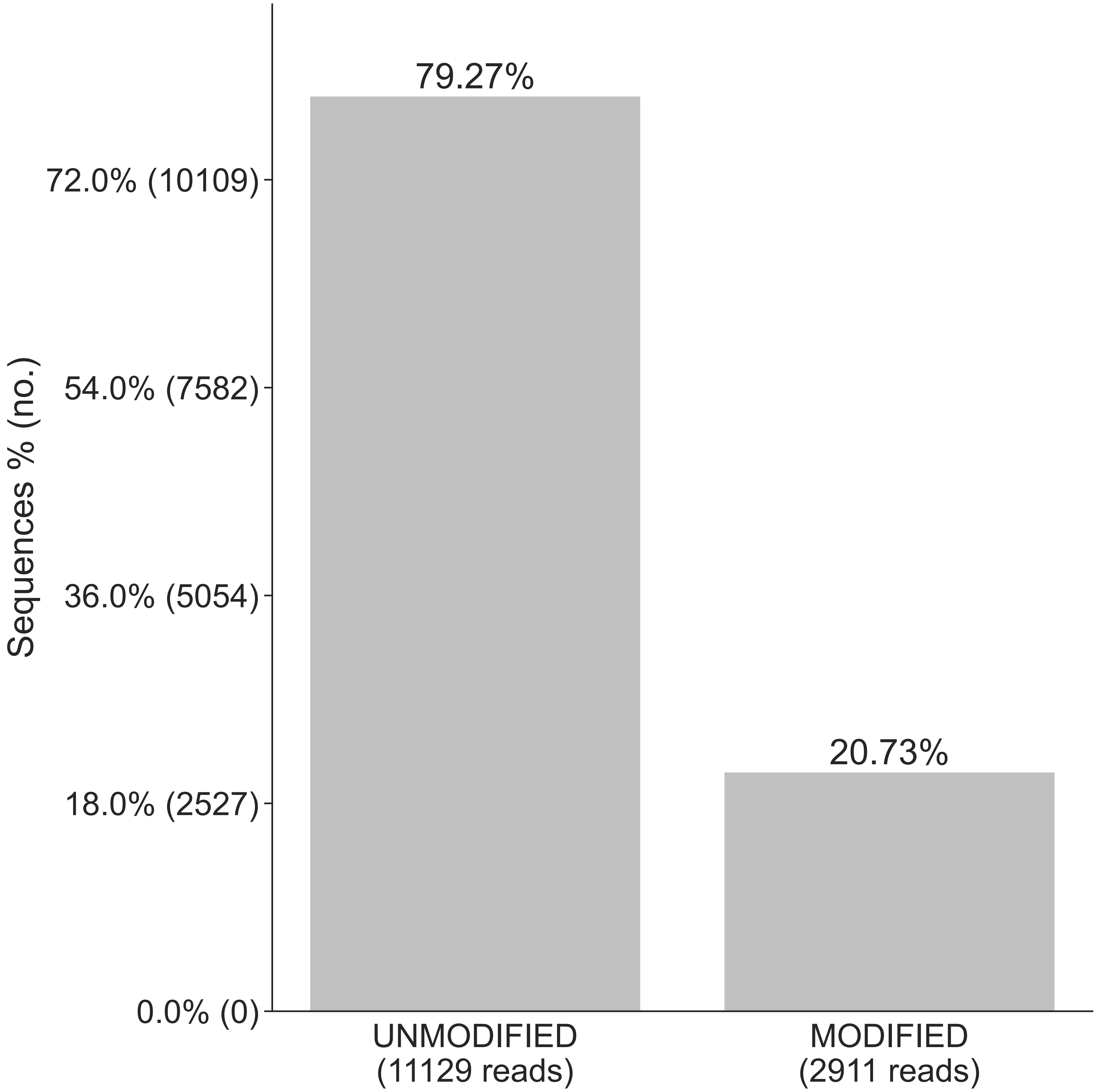
79.27%
20.73%
72.0% (10109)
54.0% (7582)
36.0% (5054)
18.0% (2527)
0.0% (0)
Sequences % (no.)
UNMODIFIED
(11129 reads)
MODIFIED
(2911 reads)
